# The role and mechanisms of miRNA in neonatal necrotizing enterocolitis

**DOI:** 10.3389/fped.2022.1053965

**Published:** 2022-11-28

**Authors:** Linghao Cai, Dengming Lai, Jiafang Gao, Hao Wu, Bo Shi, Haosen Ji, Jinfa Tou

**Affiliations:** Department of Neonatal Surgery, The Children's Hospital, Zhejiang University School of Medicine, National Clinical Research Center for Child Health, Hangzhou, China

**Keywords:** microRNA, neonatal necrotizing enterocolitis, enterocyte, inflammation, biomarker

## Abstract

Neonatal necrotizing enterocolitis (NEC), the most significant causes of neonatal mortality, is a disease of acute intestinal inflammation. At present, it is not clear exactly how the disease is caused, but it has been suggested that this disorder is a result of a complex interaction among prematurity, enteral feeding and inappropriate pro-inflammation response and bacterial infection of the intestine. A microRNA (miRNA) is a class of endogenous non-coding single-stranded RNA that is about 23 nucleotides long engaging in the regulation of the gene expression. Recently, numerous studies have determined that abnormal miRNA expression plays important roles in various diseases, including NEC. Here, we summarized the role of miRNAs in NEC. We introduce the biosynthetic and function of miRNAs and then describe the possible mechanisms of miRNAs in the initiation and development of NEC, including their influence on the intestinal epithelial barrier's function and regulation of the inflammatory process. Finally, this review aids in a comprehensive understanding of the current miRNA to accurately predict the diagnosis of NEC and provide ideas to find potential therapeutic targets of miRNA for NEC. In conclusion, our aims are to highlight the close relationship between miRNAs and NEC and to summarize the practical value of developing diagnostic biomarkers and potential therapeutic targets of NEC.

## Introduction

Neonatal necrotizing enterocolitis (NEC) is one of the most prevalent intestinal disorders affecting premature infants and is suggested to be linked to the highest rate of infants requiring surgery (1). It is noteworthy that NEC causes a significant proportion of neonatal illness and death, with a morbidity rate of 6%–10% and a death rate between 20%–30% (1–4). NEC is thought to be caused by a complex interaction among prematurity, enteral feeding and inappropriate pro-inflammation response and bacterial infection of the intestine, which lead to subsequent tissue damage (5, 6). However, the etiopathogenesis of NEC has not been completely established, which results in some difficulties in early diagnosis, prediction of prognosis and treatment strategies. The diagnosis of early clinical NEC is difficult, owing to its nonspecific clinical laboratory indicators, such as elevated levels of acute-phase proteins and pro-inflammation cytokines. Additionally, it is difficult to differentiate NEC from other neonatal conditions that have similar symptoms (7, 8). At present, treatments are non-specific and are used to achieve relief of clinical symptoms (9, 10). The increasing incidence and prevalence of NEC have produced a global medical burden (1). Hence, developing more acute clinical diagnostic, monitoring and treatment methods for NEC are urgently needed.

MicroRNA (miRNA) is a class of endogenous non-coding single-stranded RNA molecule with a length of ∼23 nucleotides that acts as a potential negative regulator (11–13). The main function of miRNA is decreasing the expression of target messenger RNAs (mRNAs) by translation inhibition or mRNA degradation *via* recognizing the 3′-untranslated regions (3′-UTRs) sequences of mRNAs (10, 14, 15). Abnormal miRNA expression has been demonstrated to play important roles in several human diseases including tumors, autoimmune diseases, cardiovascular diseases and intestinal disorders (16–19). Recently, many studies have indicated that miRNAs participate in various biological functions in bowel diseases, such as cell apoptosis, cell proliferation, intestinal epithelial barrier function, inflammation infiltration and carcinogenesis (20–24).

Many investigations have shown that miRNAs are involved in disease initiation and development, and some with pathological specificity. Therefore, changes in miRNA expression profiles have been used for early detection, diagnosis and prognosis of diseases. Zhou et al*.* summarized the functions of miRNAs in Ulcerative colitis (UC) and discussed four types of possible mechanisms miRNAs engaged in the occurrence and development of UC (25). Palangi et al*.* proposed some tissue-specific and circulating miRNAs as potential biomarkers for inflammatory bowel fast diagnosis and grade prediction (26). While different studies suggested that these miRNAs could serve as potential clinical predictors for inflammatory gastrointestinal (GI) conditions (27–29), there are few researches focusing on the roles of miRNAs in NEC. Yu et al*.* identified 16 miRNAs that displayed different expression between the NEC and control groups and verified five candidate miRNAs by RT-qPCR (7). Ng et al*.* identified miR-1290 as a promising specific biomarker that could effectively distinguish infants with NEC from sepsis (29). Wu et al*.* demonstrated that miR-431 was significantly higher (>7-fold) in NEC intestinal tissues in comparison to control tissues. The miR-431-FOXA1 axis is thought to be partially responsible for the dysregulation of the inflammatory response in NEC tissue (30). Li et al*.* indicated that miR-141-3p could mitigate necroptosis of the intestine (31).

Therefore, the aims of the present article are to review the physiological synthesis of miRNAs and to summarize the differential expression of miRNAs in NEC. We also look forward to the possibility of identifying miRNAs as novel diagnostic biomarkers or treatment targets of NEC. Furthermore, the performing mechanisms are discussed, which involve intestinal tissue barrier function, inflammatory process, potential diagnostic biomarkers and treatment interventions.

## Materials and method

### Search criteria for published studies

The literature searches in several electronic databases were conducted until December 22, 2021, including PubMed, Embase and Web of Science. We combined (using “AND”) all the search terms “non-coding RNA”, “nc RNA”, and “miRNA OR microRNA OR miR” with terms “neonatal necrotizing enterocolitis”, and “NEC”.

### Eligible studies and data extraction

After removing duplication, a total of 179 studies were obtained. Eligible studies should meet the following selection criteria: (1) original articles that assessing miRNAs expression between NEC patients or NEC animal models and healthy controls. Reviews, meta-analysis, bioinformatics, case reports, non-English articles, studies that were not miRNAs or on other diseases were excluded.

Relevant information was carefully extracted from the eligible articles presenting original data, including first author's name, year of publication, samples source, number of samples with NEC and controls, and dysregulation miRNAs identified in NEC samples.

### Ethics statement

No animal or human studies were carried out by the authors for this article.

## Results

**Figure 1:**
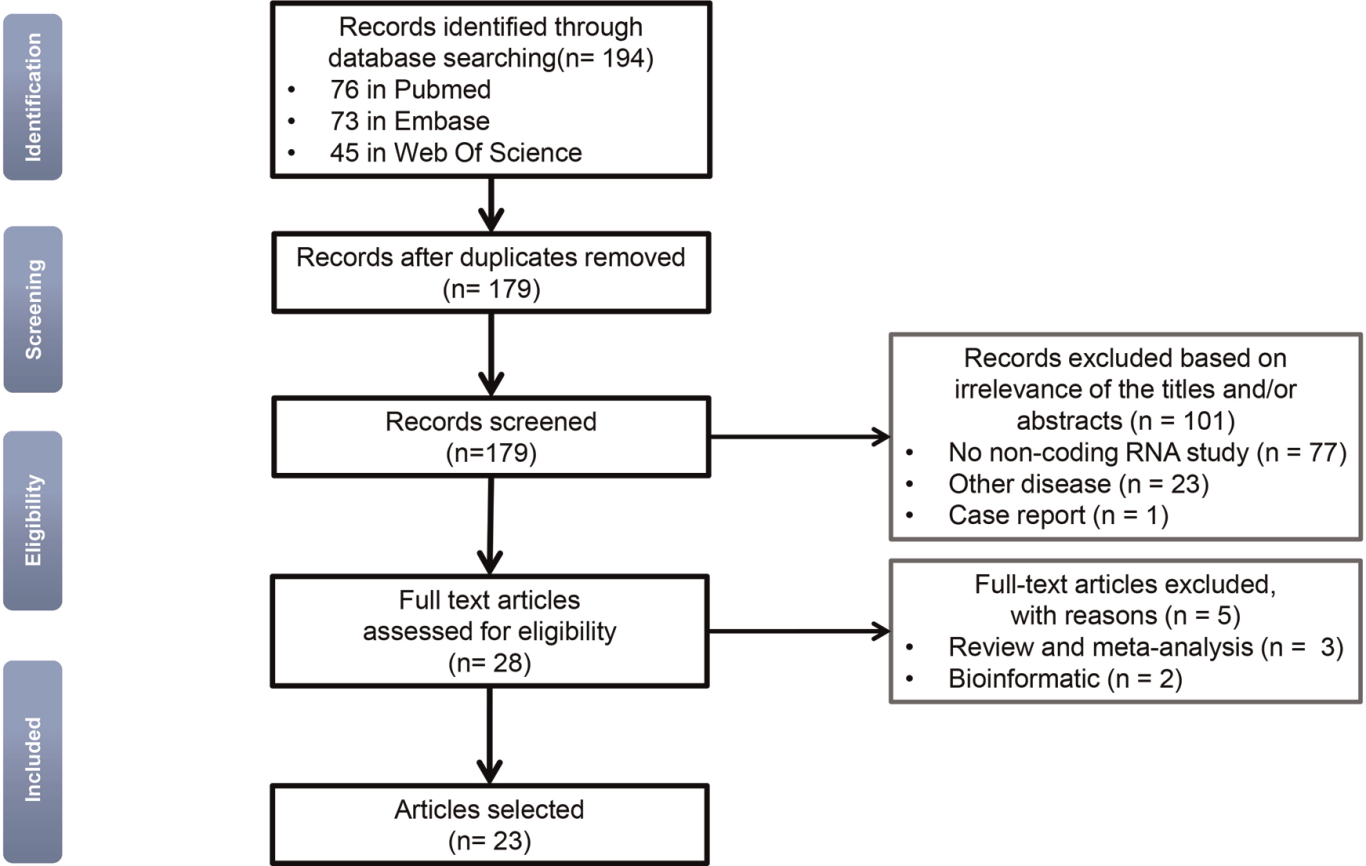
Flow-chart diagram for selection of relevant studies (n = number of records).

The flow-chart illustrates the selection of relevant studies (Figure 1). After eliminating the duplicate articles, 179 articles remained. Based on our selection criteria, the full texts of the remaining 28 publications were further retrieved and read carefully. Of these, 5 publications were excluded based on the previously defined exclusion criteria. In total, 23 publications were eventually included in this systematic review. Related information was listed in Table 1.

**Table 1:** A summary of miRNAs’ expression levels of NEC in different studies.

Study	miRNA name	Expression level	N (control)	N (NEC)	Detection method	Target gene	Sample source	Reference
Yin et al. 2019	miR-124	↑	/	/	qRT-PCR	ROCK1	Rat NEC model tissue	(32)
Sun et al. 2020	let-7d-5p	↓	40	28	qRT-PCR	LGALS3	Rat NEC model tissue	(33)
Chen et al. 2020	miR-141-3p	↓	/	8	qRT-PCR	MNX1	Rat NEC model tissue	(34)
Xia et al. 2021	miR-222	↑	12	12	qRT-PCR	C-KIT	Rat NEC model tissue	(35)
Wu et al. 2019	miR-431	↑	10	10	qRT-PCR	FOXA1	NEC human tissue	(30)
Chen* et al. 2020	miR-146a-5p	↑	22	17	qRT-PCR	ND	NEC human tissue	(36)
Wu et al. 2021	miR-223	↑	10	10	qRT-PCR	NFIA	NEC human tissue	(37)
Li et al. 2020	miR-141-3p	↓	6	6	qRT-PCR	RIPK1	NEC human serum sample	(31)
Ng et al. 2020	miR-223	↑	194	24	qRT-PCR	ND	NEC human stool sample	(38)
	miR-451a	↑				CLDN8		
Ng et al. 2019	miR-1290	↑	265	36	qRT-PCR	ND	NEC human plasma sample	(29)
	miR-1246	↑				FOXA1		
	miR-375	↑				ND		
Galley et al. 2021	miR-376a	↓	12	14	Sequencing analysis	ND	NEC human urine sample/Rat NEC model tissue	(10)
	miR-518a-3p	↓				ND		
	miR-604	↓				ND		
Yu et al. 2020	miR-27a-5p	↑	3	3	Sequencing analysis/qRT-PCR	ND	NEC human tissue/Rat NEC model tissue	(7)
	miR-187-3p	↓				ND		
	miR-219-1-3p	↑				ND		
	miR-452-5p	↑				ND		
Zhu et al. 2021	miR-34a	↑	30	30	qRT-PCR	SIRT1	NEC human serum sample/Rat NEC model tissue	(39)

↑, significantly upregulated miRNAs in NEC; ↓, significantly downregulated miRNAs in NEC.

* indicates not the same person; qRT-PCR, quantitative real-time PCR; ND, not determined.

### Overview of microRNA biogenesis and function

miRNAs a class of endogenous non-coding single-stranded RNA (approximately 23 nucleotides) that function as negative regulators by influencing target gene expression at the post-transcriptional level (22, 40). The first miRNA, Lin-4, was discovered in 1993 by Lee in the Caenorhabditis elegans (41). To date, researches have shown that approximately more than 30% to 60% of mRNAs in the human genome are modulated by miRNAs (42, 43). In the last decades, miRNAs have been largely studied to clarify their crucial role in various cellular processes, signaling pathways and physiological disorders (44–46). In 2020, as evidence of the importance of miRNAs in health and disease, there were more than 14,000 miRNA publications listed in PubMed.

It is quite clear that the biogenesis of miRNAs is complex processes including two steps: the first is to transcribe in the nucleus, and the second is to generate mature miRNAs in the cytoplasm (22, 47–49). In the first, assisted by RNA polymerase II or III synthesizes, miRNA is transcribed from the genome to primary transcript miRNA (pri-miRNA) that contains a hairpin-like structure in the nucleus (22, 50–52); then the pri-miRNA is cleaved by Drosha-DGCR8 complex into precursor miRNA (pre-miRNA) (53, 54). The pre-miRNA is further transferred from the nucleus into the cytoplasm *via* the Exportin 5 (55, 56). In the cytoplasm, the pre-miRNA is cleaved by RNase III enzyme, Dicer, in cooperation with trans-activator RNA binding protein (TRBP) into mature miRNA duplex of approximately 23 nucleotides in length (57–60). The mature miRNA duplex further separates, and the 5′ end of the miRNA strand, named the guide strand, is transferred into the Argonaute (AGO) protein to form an RNA-induced silence complex (RISC) (61–63).

**Figure 2:**
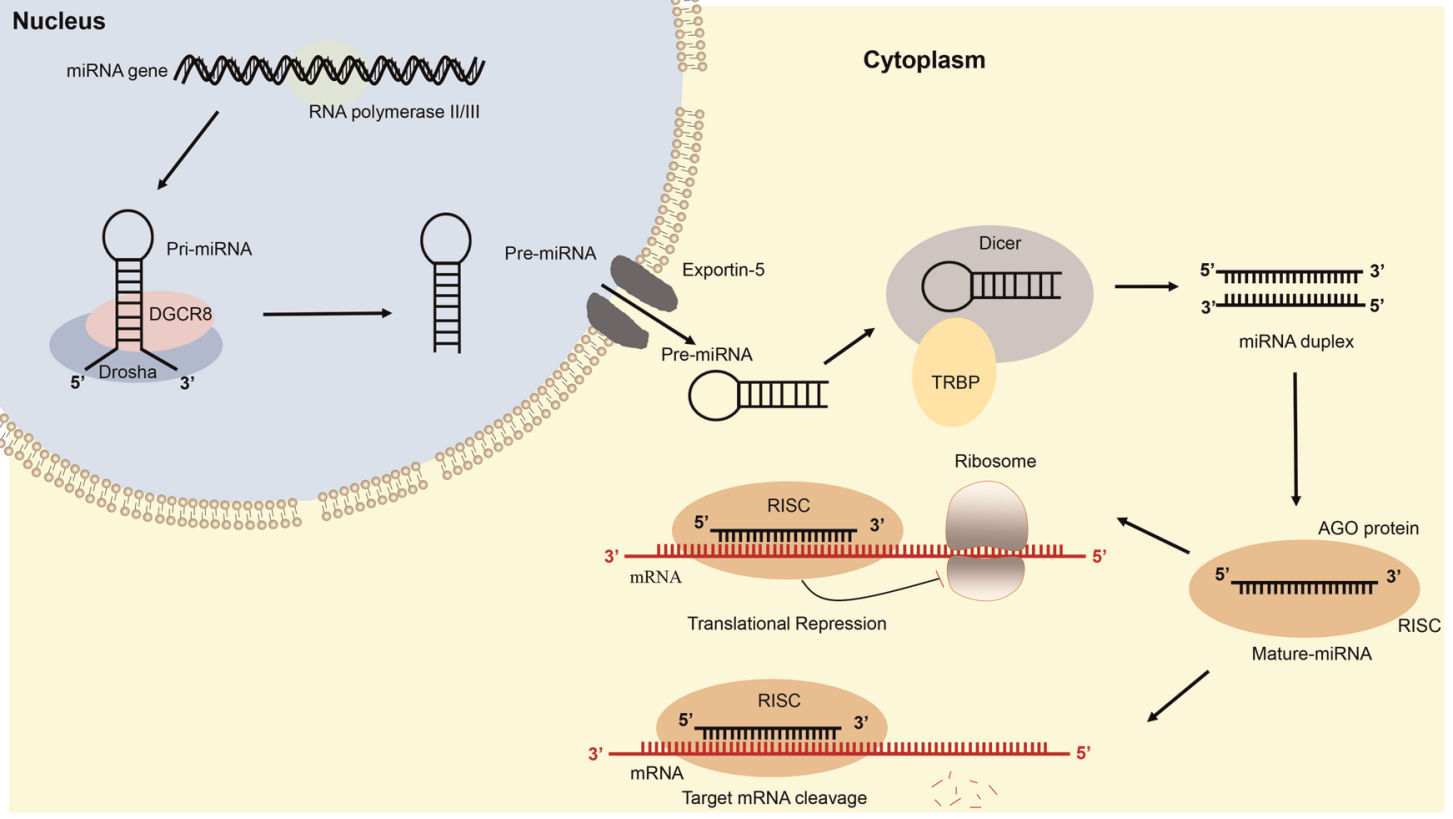
Schematic illustration of microRNA biogenesis and function.

Next, the RISC recognizes and the 3′-UTR of mRNA, resulting in degradation or translational inhibition (40, 64–66). The biogenesis and function of miRNA are shown in Figure 2.

### miRNAs involved in intestinal tissue barrier function

The intestinal epithelial cell (IEC) barrier is the most important integral part of the intestinal mucosal barrier that enables IECs to maintain immune tolerance and prevent intestinal inflammation (25, 67, 68). In the last few years, numerous studies have focused on the pathogenic mechanism of disruption of the IEC barrier. Disruption of the IEC barrier is a foremost factor that can lead to a range of inflammatory disorders, including NEC and inflammatory bowel diseases (IBD) (69, 70). Many miRNAs have been demonstrated to have a significant role in intestinal barrier function, modulating IEC growth and apoptosis as well as tight junctions among IECs (25).

A study by Yin et al*.* identified that miR-124 is upregulated and that Rho-associated coiled-coil-containing protein kinase 1 (ROCK1) and myosin phosphatase target subunit 1 (MYPT1) are downregulated in the NEC tissue, resulting in aggravating cell apoptosis of NEC tissues (32). Sun et al*.* demonstrated that the expression of Let-7d-5p is poor in intestinal tissues of NEC rats. Based on their research, they concluded that overexpression of Let-7d-5p may suppress IEC apoptosis and inflammatory factors expression in NEC (33). Li and his colleagues indicated that the miR-141-3p is downregulated in NEC patients’ serum and the *in vitro* NEC model, comparing to controls. The miR-141-3p could alleviate LPS-induced damage to IEC by inhibiting necroptosis and inflammation mediated by RIPK1 (31). miR-141-3p has also proven to be beneficial for cell viability and for prohibition of apoptosis in IEC-6 cells by directly targeting the Motor neuron and pancreas homeobox 1 (MNX1) (34). miR-431 is overexpressed in NEC tissues. miR-431 decreases the expression of epithelium tight junction regulators (HNF4A, PRKCZ) and increases apoptosis and total cell death *via* miR-431-FOXA1 axis, leading to NEC histological injury (30, 71). miR-223 is upregulated in NEC infants and inhibits the expression of target transcription factors nuclear factor I-A (NFIA). miR-223 can regulate cell proliferation, apoptosis, G protein signaling, smooth muscle contraction and inflammatory response. This pattern suggests that miR-223 may perform a vital function in cell growth and apoptosis, as well as in epithelial barrier permeability and tight junction integrity (37). Several other articles have reported that miR-223 is induced by the IL-23/Th 17 pathway and does damage to the IECs tight junctions by targeting Claudin-8 (23, 25, 72). miR-1290 is significantly higher in NEC patients (29). It affects the expression of Claudin-1, Zo-1 and MLCK, which are essential component of the epithelial barrier (73).

**Figure 3:**
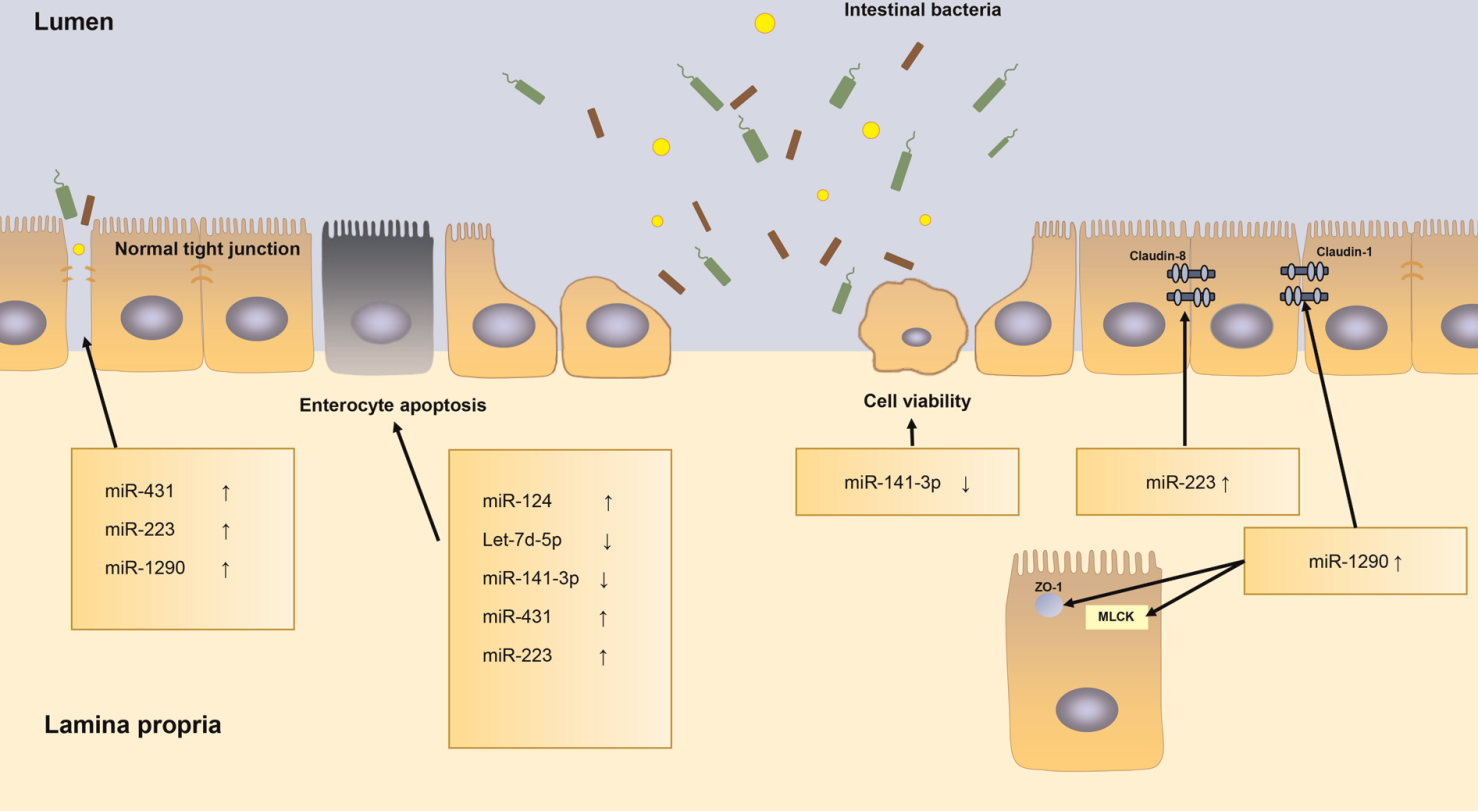
The roles of miRNAs in regulating intestinal barrier function.

To summarize, miRNAs have a major effect on maintaining the intestinal barrier function (Figure 3). miRNAs can regulate the tight junctions among IECs, can alter their permeability, and are concerned with cell apoptosis.

### miRNAs involved in pro-inflammatory and anti-inflammatory process

NEC is a gastrointestinal emergency characterized by severe inflammation storm (36). IECs are invaded by abnormal bacteria, causing tissue damage and inflammation, which result in systemic and intestinal complications (31, 74). Various studies have demonstrated that activation of Toll-like receptor 4 (TLR4) and NF-kB signaling pathways are critical in modulating the expression of pro-inflammatory cytokines in different cell types (75). Additionally, the TLR4/NF-kB signaling pathways are well-known to promote the progression of NEC (76, 77).

Toll-like receptors (TLRs) play an important part in the innate immune system by detecting specific molecular patterns of the pathogenic microorganisms. NF-kB activation is enabled by TLR-4 stimulation, leading to the release of inflammatory cytokines, IFN-*β* and IFN-inducible proteins (26). Several researches have shown that dysregulation of miRNAs acts as a crucial regulator in inflammatory diseases such as IBD that have a major impact on TLRs and its associated signaling pathways (22, 23, 25, 26, 78). However, only limited studies have concentrated on the influence of miRNAs on the inflammatory process of NEC. Therefore, some possible mechanisms of miRNAs' involvement in the inflammatory progression of NEC disease are summarized here.

A study by Wu et al*.* showed that the miR-431-FOXA1 axis might enhance the inflammatory response in NEC tissues by reducing the expression of Phospholipase A2 Group IIA (PLA2G2A) and by increasing the expression of IL-6, IL-8, IL-10, NFKB2 and TNF (30). MiR-223 has been considered to act both anti-inflammatory and pro-inflammatory functions in other diseases (79, 80). Wu et al*.* observed that miR-223 upregulated the expression of IL-6 and IL-8 in FHs74 model (37). Zhu et al*.* found that miR-34a was notably upregulated in NEC, which could promote the production of inflammatory cytokines (TNF-α, IL-1β, IL-6, IL-8) and anti-inflammatory cytokines IL-10 by downregulating sirtuin1 (SIRT1) expression (39). Xia et al*.* indicated that C-kit expression was suppressed by TNF-α *via* miR-222 upregulation resulting in an increased inflammatory response (35).

Let-7d-5p is thought to be an anti-inflammatory miRNA. Sun et al*.* demonstrated that Let-7d-5p inhibits expression of its target gene galectin3 (LGALS3), consequently regulating the TLR4/NF-kB signaling pathway and decreasing proinflammatory cytokines (TNF-α, IL-1β, IL-6) in NEC neonatal rats (33). MiR-141-3p protects IECs by suppressing inflammation mediated by RIPK1 and by reducing the expression of IL-6 and TNF-α in LPS-treated CaCo-2 cells (31). Chen et al*.* also found that another miR-141-3p target gene Motor neuron and pancreas homeobox 1 (MNX1) can override the effects of miR-141-3p in LPS-damaged IECs (34). Recent researches show that NLRP3 inflammasome plays a role in NEC development (81, 82). MiR-146-5p can attenuate inflammation and intestinal injury by inhibiting downstream inflammatory factors of the NLRP3 inflammasome and CLIC4 membrane expression in NEC (36).

**Figure 4:**
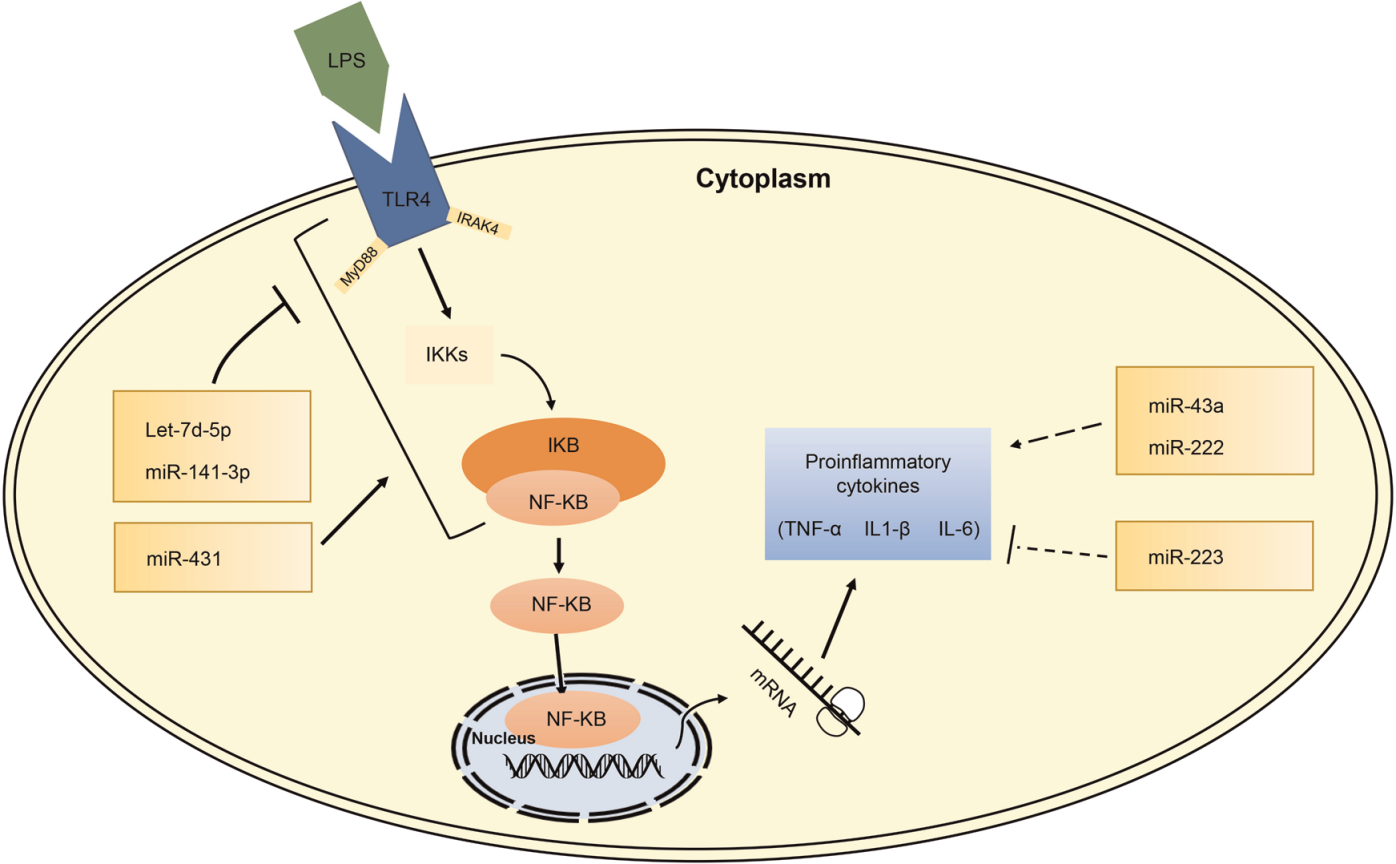
miRNA dysregulation of inflammatory process in NEC pathogenesis.

In general, large amounts of researches have shown that miRNAs induction and repression can affect some biological processes and result in inflammation (Figure 4). The dysregulation of miRNAs which leads to activation or inhibition of signaling pathways might reflect some underlying inflammatory processes in NEC.

### miRNAs as potential biomarkers for diagnosis in NEC

**Figure 5:**
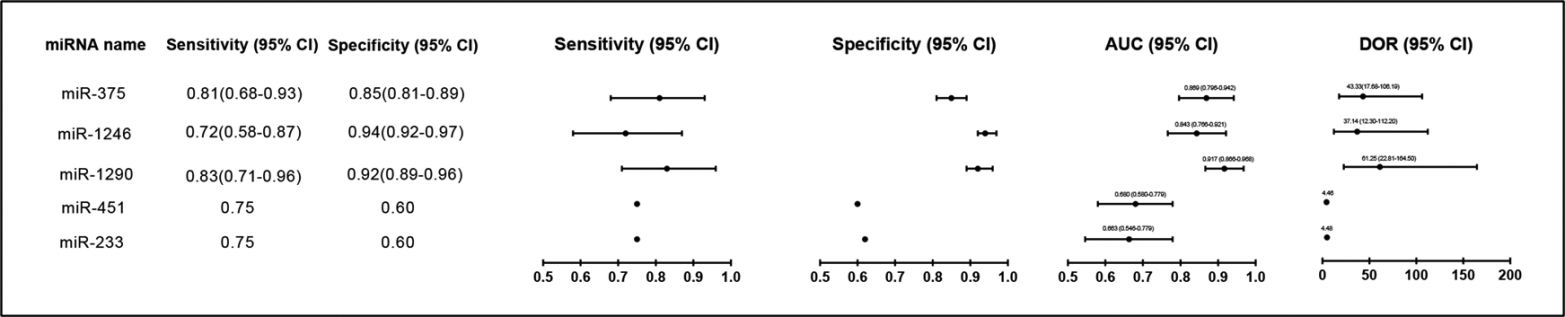
Forest plot of miRNAs levels as biomarkers for diagnosis of NEC patients in different studies. AUC, Area Under Curve; DOR, diagnostic odds ratio; 95% CI, 95% confidence interval.

Early diagnosis of NEC can help in timelier and more effective treatment and can avoid disease progression. At present, NEC diagnosis has always been a serious challenge. The early symptoms of the disease are concentrated in the gastrointestinal tract, including feeding intolerance and abdominal distension (83). However, these symptoms are nonspecific in the early stage and can easily be misdiagnosed as non-NEC disorders, such as functional gastrointestinal motility disorders in prematurity or sepsis- or electrolyte-induced ileus (38, 84, 85). When the diagnosis is delayed or missed, the patient may experience fulminant clinical deterioration, as well as septic shock, disseminated intravascular coagulation, and even die within a few hours or days (38, 86). Therefore, finding new diagnostic biomarkers for early diagnosis of NEC is urgent and will allow the initiation of corresponding treatment plans. miRNAs that are chemically stable in biofluid have the potential as non-invasive biomarkers of disease (87, 88). At present, many studies offer the prospect of miRNA as a non-invasive diagnostic method for IBD (19, 23, 25, 87). However, only a few articles have explored the usefulness of miRNA in the diagnosis of NEC (Table 2). Therefore, in the present article, we briefly summarize the latest findings and try to pay more attention to miRNAs that have been validated in NEC clinical populations (Figure 5).

**Table 2:** miRNAs levels as biomarkers for diagnosis of NEC patients in different studies.

miRNA name	Sample type	Disease cohort	Association	Expression states	Sensitivity	Specificity	AUC (95% CI)	DOR (95% CI)	Reference
miR-233	Stool sample	24 NEC vs. 194 non-NEC infants	Diagnosis of NEC	↑	0.75	0.62	0.663 (0.546–0.779)	4.84	(38)
miR-451	Stool sample			↑	0.75	0.6	0.680 (0.580–0.779)	4.46	
miR-1290	Plasm sample	36 NEC vs. 265 non-NEC infants		↑	0.83	0.92	0.917 (0.866–0.968)	61.25 (22.81–164.50)	(29)
miR-1246	Plasm sample			↑	0.72	0.94	0.843 (0.766–0.921)	37.14 (12.30–112.20)	
miR-375	Plasm sample			↑	0.81	0.85	0.869 (0.796–0.942)	43.33 (17.68–106.19)	
miR-376a	Urine sample	9 surgical NEC vs. 4 non-NEC sepsis		↑	/	/	/	/	(10)
miR-518-3p	Urine sample	7 surgical NEC vs. 4 non-NEC sepsis		↑	/	/	/	/	
miR-604	Urine sample	14 NEC vs. 8 control		↑	/	/	/	/	

AUC, Area Under Curve; DOR, diagnostic odds ratio; 95% CI, 95% confidence interval.

The day of clinical presentation of NEC was defined as Day 0 in the study by Ng et al*.* They collected a total of 17 stool samples (Day 0) and 7 samples (Day 1–2) for analysis. The analysis of miRNAs expression profiles in stool samples of 24 NEC infants and 194 non-NEC infants (controls) indicated that levels of miR-223 (10.5-fold, *p *= 0.008) and miR-451 (4.5-fold, *p *= 0.01) were significantly higher in NEC infants compared to the controls. Areas under receiver operating characteristic of miR-233 and miR-451a were 0.663 (*p *= 0.009) and 0.680 (*p *= 0.004), respectively, with specificities of 0.62 and 0.60, respectively, and with a sensitivity of 0.75. In addition, these two fecal miRNAs have also been shown to have positive predictive values (PPVs) that range between 0.19 and 0.20. The PPVs could be greatly increased when miRNA and CRP (Day 1) were used in combination (38). Ng et al*.* also investigated the plasma miRNA expression profiles in the NEC compared to sepsis group and non-NEC/non-sepsis group in a case-control study and found that miR-1290, miR-1246 and miR-375 were increased. The ROC curves of miR-1290, miR-1246 and miR-375 were further validated in a cohort study (*n* = 301) with 0.92, 0.84 and 0.87, respectively. Among these curves, plasm miR-1290 (Day 0) is considered to have the most reliable diagnostic utility in determining both medical and severe surgical NEC (29). Galley et al. collected urine from patients at three time points: (1) within 24 h of admission (immediately after diagnosis), (2) 2–5 days of admission (during disease), and (3) 6–14 days after admission (during disease resolution). As revealed by transcriptomic analysis of urine-derived miRNAs, they observed that miR-376a, miR-518-3p and miR-604 were considerably changed in NEC compared to non-NEC sepsis and healthy controls. However, these miRNAs need to be further validated in larger sample sizes (10).

In summary, many miRNAs have been found to have predictive diagnostic value in other diseases, such as IBD. However, the predictive role of miRNAs in NEC is still rarely studied and needs to be verified by a large clinical sample size.

### miRNAs as potential therapies in NEC

Earlier, we described the importance of miRNAs in various mechanisms of NEC and their diagnostic role in NEC. Given this close relationship, it is not difficult to conclude that miRNAs also may have a significant part in the treatment of NEC. Currently, miRNAs-related therapeutic interventions have been widely discussed in various diseases, such as tumors and IBD (24, 89–91). miRNA-based therapies generally contain two basic approaches: miRNA antagonism and mimicry (92). miRNA antagonists can inhibit the down-regulation of pathological target genes caused by physiological miRNA overexpression, whereas miRNA mimics can restore pathological up-regulation of target genes caused by physiological miRNA under-expression (19, 92). miRNA antagonists can be one of three things: anti-RNA oligonucleotides (AMOS), miRNA sponges and miRNA masks. miRNA mimics include synthetic oligonucleotides and miRNA expression gene vectors (22). Polytarchou et al*.* revealed high miR-214 expression in the colon tissues of patients with active ulcerative colitis (UC) or colitis-associated colon cancer and its association with disease progression. miR-214 inhibitors could significantly reduce inflammation and tumor number and size (93). Research by Guo et al*.* indicated that the inhibition of miR-7 can improve the clinical symptoms of IBD model mice and can reduce the mucosal pathological damage in the intestine, which has a protective role in IBD treatment (94).

Yin et al*.* showed that overexpression of miR-124 significantly inhibited the expressions of ROCK1, MYPT1 and TLR9 in IEC-6 cells. Apoptosis and inflammatory infiltration of intestinal cells are alleviated by inhibiting miR-124 *via* its target ROCK1 and its downstream molecules (32). A study by Sun et al*.* indicated that miRNA Let-7d-5p has been described as anti-inflammatory. In comparison with the NEC-control and NC mimic groups, the Let-7d-5p mimic group had a significantly lower apoptosis rate and inflammatory factors expression (33). The expression level of Forkhead Box A1 (FOXA1) was decreased in NEC tissues and in Caco-2 cells treated with miR-431 mimic. Targeting FOXA1 and its tissue-specific binding miRNAs like miR-431 has also been considered as a novel approach for NEC therapy (30). MiR-141-3p mimics can reduce inflammation and necrosis mediated by Receptor Interacting Serine/Threonine Kinase 1 (RIPK1) (31). Similarly, Chen et al*.* demonstrated that miR-141-3p mimic can inhibit the expression of inflammatory factors through miR-141-3P-MNX1 axis (34). MiR-146a-5p is remarkably significantly overexpressed miRNA in both NEC human and mouse intestinal tissues. NEC mouse model can improve survival rate and mitigate NEC-induced weight loss and intestinal injury by adenovirus transduction (36). Nuclear Factor I A (NFIA) was considered as a target of miR-223. Inhibition of miR-233/NFIA axis may provide new ideas for the treatment of NEC (37). TNF-α typically activates genes involved in inflammation. Xia et al*.* found that TNF-α up-regulated miR-222 expression and inhibited c-kit expression. Antagonists of miR-222 can reverse the decrease of c-kit expression (35). Zhu et al*.* found that intestinal villi injury can be ameliorated by miR-34a inhibitors and SIRT1 activators (39).

In this section, we summarize the latest research on the potential treatments of NEC diseases with miRNA (Table 3). However, the potential therapeutic approaches are yet to be investigated and proved with more biochemical analysis and clinical trials. It is hoped that these findings will offer us new ideas for the treatment of NEC in the future. However, their mechanisms and clinical effects remain to be further verified.

**Table 3:** Summary of potential therapeutic miRNAs in NEC.

miRNA name	Targets	Functions	Therapy	Reference
miR-124	RCOK1	Involves in apoptosis and inflammatory cell infiltration	Antagonism	(32)
Let-7d-5p	/	Involves in apoptosis and inflammatory factors expression	Mimicry	(33)
miR-431	FOXA1	Involves in inflammatory response in NEC tissues	Antagonism	(30)
miR-141-3p	RIPK1	Involves in inflammation and necrosis	Mimicry	(31)
miR-141-3p	MNX1	Involves in inflammatory factors expression	Mimicry	(34)
miR-146a-5p	/	Involves in NEC rat survival, weight loss and intestinal injury	Mimicry	(36)
miR-223	NFIA	Involves in inflammation and cellular functions	Antagonism	(38)
miR-222	C-KIT	Involves in inflammation	Antagonism	(37)
miR-34a	SIRT1	Involves in intestinal villi injury	Antagonism	(39)

## Discussion

miRNAs are short non-coding RNAs, whose dysregulation plays a crucial part in NEC disease. Considerable evidence demonstrated that miRNAs profiles are altered in NEC patients. Dysregulation of miRNA may be closely involved in the genesis and development of NEC disease. Based on these findings, miRNAs are also expected to be novel, reliable, non-invasive diagnostic markers and potential therapeutic targets for NEC. In this article, we provided a systematic review of the important role of various miRNAs in the pathogenesis of NEC, including regulation of tight junctions and intestinal barrier function, apoptosis and inflammatory response. We also summarized some current researches in the diagnosis and treatment of miRNAs in NEC.

Current studies on miRNA in NEC mainly concentrate on three aspects. The first aspect is about the specific mechanism in which miRNAs are involved. It mainly involves impaired intestinal barrier function, dysregulation of apoptosis and inflammatory infiltration. At present, despite numerous of researches about specific mechanisms, most articles focus solely on one miRNA or one pathway. Among them, TLR4/NFKB pathway receives the most concern, but there are no studies that pay attention to the link between them and other signaling pathways. The construction of a regulatory network from miRNA to its target genes and other miRNAs is worthy of further study in the future.

The second aspect concerns the abnormal expression of miRNAs in NEC. Many miRNAs have been shown to be upregulated or downregulated in NEC. Abnormal expression of miRNAs in NEC provides the possibility to select diagnostic biomarkers of NEC. Ng et al*.* demonstrated that plasma miR-1290 is a novel and specific biomarker. miR-1290 could effectively distinguish NEC (both medical and surgical NEC) from other diseases with similar symptoms, such as neonatal sepsis or neonatal enteritis. They also established a signature of miR-1290 at the onset of NEC (Day 0) and CRP 24 h later (Day 1) for optimizing diagnosis. The combination of miR-1290 and CRP together facilitate neonatologists to detect NEC more quickly and initiate treatment (29). Analysis by Galley et al*.* indicated that urine miR-376a, miR-518a and miR-604, could be used for differential diagnosis of NEC and non-NEC sepsis or healthy individuals (10). miR-223 and miR-451a in stool have also been identified as specific non-invasive diagnostic markers for NEC (38). Several different miRNAs have been combined as a diagnostic marker in IBD, but not in NEC (95, 96). The combination of multiple miRNAs and miRNAs with some previous NEC biomarkers may improve diagnostic accuracy. In addition, these articles are all concerned with the diagnostic effects of non-invasive miRNAs. Plasma, urine and feces from patients are relatively easy to obtain. However, these articles also have some shortcomings. First, the diagnosis of NEC is inherently uncertain, and the time taken to collect plasma from patients diagnosed with NEC may have an error in the time of diagnosis (29). Fecal sample extraction also may be affected by the intestinal function of patients in NEC, resulting in poor applicability. Second, the sample size of these studies is too small, and larger clinical sample sizes are necessary for validation in order to achieve the identification of specific diagnostic biomarkers for NEC. Furthermore, different stages in the development of NEC may have different subsets of miRNA with different levels, even the same miRNA may increase or decrease at different stages. Most of the current studies focus on comparing the overall miRNA expression differences between NEC patients and control patients. Nevertheless, only a few of them analyze individual miRNAs over different phases of NEC development. Most of the samples were usually collected within 24 h of the onset of the clinical presentation of NEC. Therefore, miRNA expression levels cannot be continuously tracked. It may also be due to the difficulty of consistently obtaining samples. We believe that it is worthy of attention in conducting similar studies in the future.

The third aspect is to explore possible therapeutic miRNA targets. Many miRNAs have been implicated in the genesis and development of NEC, providing potential therapeutic strategies. One miRNA can participate in the regulation of hundreds of proteins. There is no way to avoid the problem of non-target proteins being affected by miRNA therapy (97). In our article, we summarize some of the currently published studies on miRNAs in the treatment of NEC. However, the potential therapeutic approaches are yet to be investigated and proved with more biochemical analysis and clinical trials. Additionally, there are still some shortcomings worth thinking about. At present, most of the researches on the treatment of miRNAs are still in the stage of cell experiments. Whether different cell types have a different impact on the therapeutic effect, how to design the antagonists and mimics of miRNAs, and how to maximize the therapeutic effect and reduce side effects are topics for additional consideration and research.

In conclusion, miRNAs have an important part in NEC. It is possible to use miRNAs as diagnostic biomarkers or therapeutic targets. It is hoped that the practical application of miRNAs in clinical practice can be further validated in future studies.
